# Efficacy of Endoscopic Radiofrequency Ablation for Treatment of Reflux Hypersensitivity: A Study Based on Rome IV Criteria

**DOI:** 10.1155/2022/4145810

**Published:** 2022-03-27

**Authors:** Yuan-Xi Jiang, Zhi-Yu Dong, Jun-Wen Wang, Ying Chen, Hui-Hui Sun, Shu-Chang Xu

**Affiliations:** Department of Gastroenterology, Tongji Hospital, Tongji University School of Medicine, Shanghai, China

## Abstract

**Objective:**

Effective therapies for reflux hypersensitivity are lacking. Endoscopic radiofrequency ablation may reduce the sensitivity of the distal esophagus through direct interference with nociceptors or vagal afferent fibers and thus may be useful in reflux hypersensitivity. The aim of this study is to assess the effectiveness and possible mechanisms of endoscopic radiofrequency ablation in reflux hypersensitivity patients.

**Methods:**

Patients with reflux hypersensitivity who fulfilled the Rome IV criteria and who wished to receive further treatment were recruited. Endoscopic radiofrequency ablation was delivered to the gastroesophageal junction. Data were collected by questionnaire using a 6-point Likert scale. The primary outcome measure was effect on symptoms including heartburn, regurgitation, and chest pain. The secondary outcomes were degree of satisfaction, medication use, acid exposure time (AET), low esophageal sphincter (LES) pressure, and total reflux episodes. We also assessed positive cell density of transient receptor potential vanilloid type 1 receptor (TRPV1) and calcitonin gene-related peptide (CGRP), both of which are biomarkers of afferent fibers, in biopsies obtained from esophageal mucosa 0.5 cm-1 cm above the Z line. These scales will be administered at baseline, 3-month follow-up, 6-month follow-up, and 12-month follow-up.

**Results:**

A total of 22 reflux hypersensitivity patients were enrolled (14 males, median age 50.0 years). A significant improvement in symptom scores (heartburn, regurgitation, and chest pain) was noted at 3 months, 6 months, and 12 months (*P* < 0.001). Satisfaction with life increased to 72.7% (16/22), 72.7% (16/22), and 68.2% (15/22) at 3, 6, and 12 mo, respectively, compared with baseline (*P* < 0.001). Nineteen patients reduced their medication use after treatment. Of these, 22.7% (5/22), 31.8% (7/22), and 40.9% (9/22) subjects stopped medication use at 3 mo, 6 mo, and 12 mo, respectively. No statistical differences were noted in AET, LES pressure, or total reflux episodes from preoperation to 12 mo postoperation. After treatment, the positive cell density of both TRPV1 and CGRP decreased significantly; however, only TRPV1 had a positive correlation with heartburn (*r* = 0.51, *P* = 0.03) and chest pain (*r* = 0.77, *P* < 0.01).

**Conclusion:**

Endoscopic radiofrequency ablation was an effective and safe therapeutic option in reflux hypersensitivity patients. Further studies with large sample size are required to validate the role of radiofrequency in reflux hypersensitivity.

## 1. Introduction

Reflux hypersensitivity refers to patients with esophageal symptoms who lack evidence of reflux on endoscopy or abnormal acid exposure on reflux monitoring, but the symptoms are related to physiologic reflux [1]. Pain modulators and proton pump inhibitors (PPIs) were considered the major modalities of treatment [1–4]; however, therapeutic trials with these agents remain empiric. There is very limited evidence on endoscopic antireflux procedures in patients with reflux hypersensitivity based on the Rome IV criteria [1].

Endoscopic radiofrequency ablation of the lower esophageal sphincter (LES) is an effective treatment for GERD, which can improve GERD symptoms by delivering radiofrequency energy to the LES, leading to increased basal pressure and reduced compliance [5–7]. It has also been suggested that the procedure might reduce the sensitivity of the distal esophagus through direct interference with nociceptors or vagal afferent fibers [8–10]. Therefore, radiofrequency ablation may be effective for reflux hypersensitivity by reducing peripheral sensitization, which is considered one of the main underlying mechanisms in patients with reflux hypersensitivity. The transient receptor potential vanilloid type 1 receptor (TRPV1) and calcitonin gene-related peptide (CGRP) have been widely used as markers of nociceptive afferent nerves, which are important in sensory sensitization [11–14]. Whether TRPV1 and CGRP play an important role in the treatment of reflux hypersensitivity is worth exploring. In addition, the correlation between TRPV1/CGRP and the severity of reflux hypersensitivity symptoms remains to be investigated.

We therefore recruited a prospectively followed cohort of reflux hypersensitivity patients to investigate the efficacy of radiofrequency ablation in reflux hypersensitivity and to determine the potential role of TRPV1 and CGRP in the development and treatment of reflux hypersensitivity.

## 2. Materials and Methods

### 2.1. Participants

We conducted a single-arm cohort study of radiofrequency energy to the gastroesophageal junction for the treatment of patients with reflux hypersensitivity from July 2018 to April 2020. The study protocol was approved by the Ethics Committee of Tongji Hospital, and all patients signed an informed consent.

**Figure 1:**
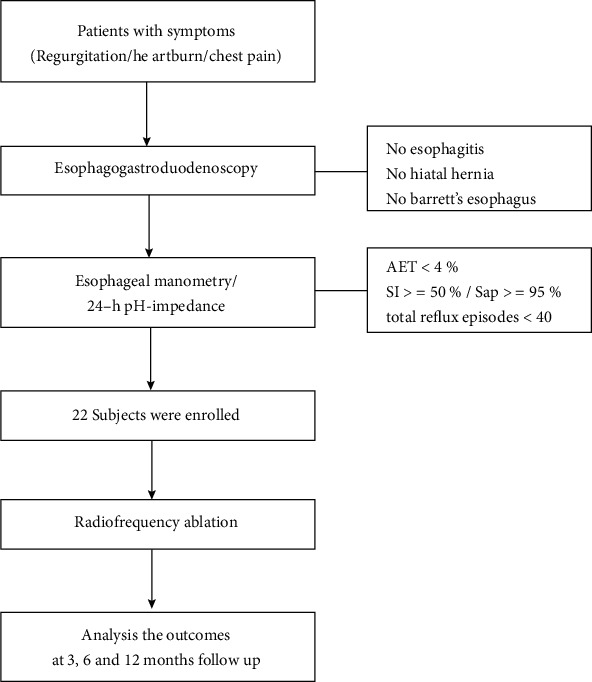
Study flow diagram.

A total of 22 reflux hypersensitivity patients were enrolled in this study. The trial flow diagram is presented in Figure 1. Potential participants were recruited from the medical practices and the general population at Shanghai Tongji Hospital, a tertiary general hospital in Shanghai, China. Participants met the following criteria: (1) presence of GERD-like symptoms (regurgitation and/or heartburn and/or chest pain) at least 6 mo prior to diagnosis with a frequency of at least twice a week; (2) age > = 18 years; (3) 24-h pH-impedance study (off medications) showing normal esophageal acid exposure (acid exposure time (AET) <4%) and positive symptom reflux association (symptom index (SI) > = 50% or symptom association probability (SAP) > = 95%); (4) total reflux episodes <40 per 24 h; (5) esophageal manometry showing normal esophageal peristalsis and sphincter relaxation; and (6) esophagogastroduodenoscopy (EGD) showing no esophagitis, no hiatal hernia, and no Barrett's esophagus. All patients stopped their drugs for 1 week before the gastroscopy and pH-impedance monitoring.

Participants with coagulation disorders, cardiogenic chest pain, prominent dysphagia, previous esophageal or gastric surgery, psychiatric disorders, or unstable disorders were excluded.

### 2.2. Intervention

**Figure 2:**
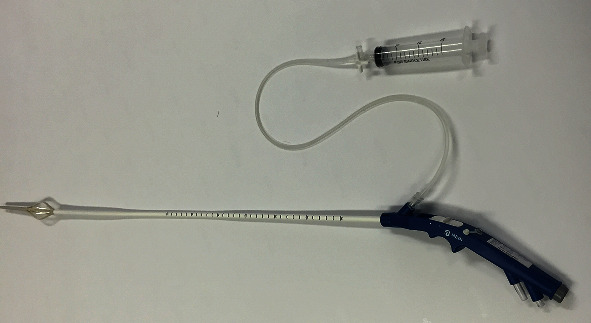
Radiofrequency catheter.

**Figure 3:**
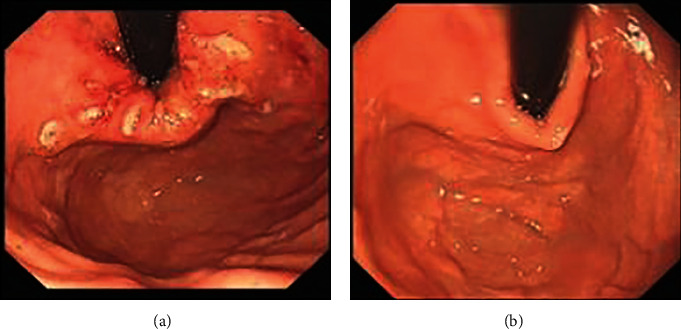
Endoscopic image immediately (a) and 12 months (b) after the procedure.

Before ablation, one biopsy was taken from 0.5 cm to 1 cm above the gastroesophageal junction. A Chinese self-developed radiofrequency ablation device, MER-200GA, was used to perform the therapeutic procedures (Figure 2). During the sedated EGD, the endoscopists measured the distance to the gastroesophageal junction, withdrew the endoscope, and introduced the radiofrequency delivery catheter orally. The catheter consisted of a flexible balloon basket with 4 electrode needle sheaths (diameter, 22 mm; length, 5.5 mm). The endoscopists inflated the balloon 1.5 cm proximal to the gastroesophageal junction and delivered the radiofrequency energy for 1 min. The catheter was then rotated 45°, and the procedure was repeated. This process was serially repeated every 0.5 cm, covering the area from 1.5 cm above to 1.5 cm below the gastroesophageal junction. The other 6 sets of this process were also used at the gastric cardia level and 0.5 cm above it, with the needle rotated 30° each time. Finally, a total of 80 lesions were treated at nine levels. Then, a posttreatment EGD was performed to assess the lesion placement (Figure 3).

Data were collected by questionnaire using a 6-point Likert scale. The frequency was graded as 0 (none), 1 (less than once a week), 2 (once or twice a week), 3 (three or four times a week), 4 (five or six times a week), and 5 (more than six times a week). The severity was graded as 0 (none), 1 (slight), 2 (mild), 3 (moderate), 4 (severe), and 5 (extremely severe). The total of the frequency score and the severity score for each of these measures were designated as the symptom score.

### 2.3. Outcome Measures

The primary outcomes were symptoms which include heartburn, regurgitation, and chest pain. The secondary outcomes were degree of satisfaction with life quality (satisfied, acceptable, and dissatisfied), medication use (using a questionnaire), and the indicators of 24-h pH-impedance monitoring and esophageal manometry including AET, LES pressure, total reflux episodes, and positive cell density of TRPV1 and CGRP, both of which are biomarkers of afferent fibers, in biopsies obtained from esophageal mucosa 0.5 cm-1 cm above the gastroesophageal junction.

### 2.4. Follow-Up

At 3, 6, and 12 mo after treatment, the symptoms, satisfaction with radiofrequency treatment, and medication use were evaluated and compared with pretreatment. At 12 mo, 24-h pH-impedance monitoring, esophageal manometry, and EGD with biopsies were also performed. The biopsies obtained from distal esophageal mucosa were immune-stained with the primary antibody against TRPV1 and CGRP. The AET, LES, pressure, total reflux episodes, and positive cell density of TRPV1 and CGRP at 12 mo after treatment were compared with those pretreatment.

### 2.5. Statistics and Sensitivity Analysis

We compared 2 different methods for evaluating missing data. The complete method evaluated only subjects with complete data for a given outcome. The carryforward method evaluated all subjects after carrying forward the last available value for missing data.

For variables with normal distribution, we reported mean ± standard deviation (SD) and performed comparisons using the paired Student *t*-test. For variables without normal distribution, we reported median values and performed comparisons using the Wilcoxon-matched pairs signed-rank test.

Pearson's correlation test was used to evaluate the relationship between symptoms and density of afferent fibers in the distal esophagus. For all correlation analyses, we considered an absolute value of the coefficient below 0.3 as a weak correlation, 0.3 to 0.5 as a moderate correlation, and above 0.5 as a strong correlation, as recommended by Cohen.

All reported *P* values were two-sided with *P* < 0.05 defined as statistically significant. All analyses were performed using R (R Foundation for Statistical Computing, Vienna, Austria).

## 3. Results

A total of 22 reflux hypersensitivity patients were enrolled in the study; the baseline characteristics of the patients are outlined in Table 1. The mean duration of the radiofrequency procedure was 40.5 min, and the average hospitalization period was 2.4 days.

### 3.1. Primary Outcomes: Symptoms

**Figure 4:**
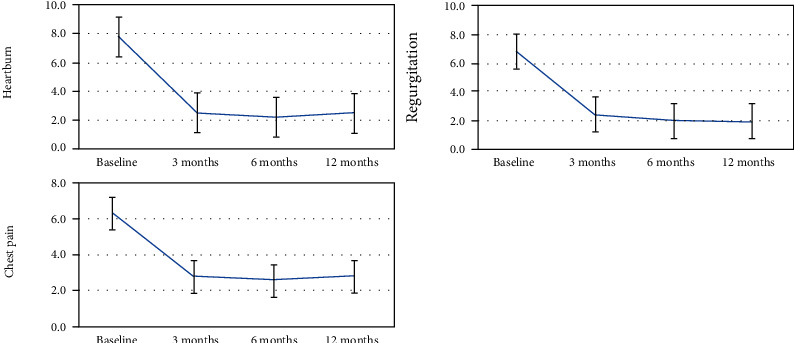
Symptoms' changes after radiofrequency procedure. Significant reductions in symptom scores (heartburn, regurgitation, and chest pain) at 3 months, 6 months, and 12 months.

The radiofrequency treatment significantly improved heartburn, regurgitation, and chest pain scores at 3, 6, and 12 mo after treatment (at 3 mo: mean decrease in heartburn, −5.3 ± 1.1, *P* < 0.001; mean decrease in regurgitation, −4.4 ± 1.2, *P* < 0.001; mean decrease in chest pain, −3.5 ± 1.1, *P* < 0.001; at 6 mo: mean decrease in heartburn, −5.6 ± 1.3, *P* < 0.001; mean decrease in regurgitation, −4.8 ± 1.5, *P* < 0.001; mean decrease in chest pain, −3.7 ± 1.4, *P* < 0.001; and at 12 mo: mean decrease in heartburn, −5.3 ± 1.3, *P* < 0.001; mean decrease in regurgitation, −4.9 ± 1.4, *P* < 0.001; mean decrease in chest pain, −3.5 ± 1.0, *P* < 0.001) (Table 2, Figure 4).

### 3.2. Secondary Outcomes

#### 3.2.1. Degree of Satisfaction with Life Quality and PPIs Usage

The radiofrequency treatment significantly improved the degree of satisfaction with life quality and medication use at 3, 6, and 12 mo after treatment (*P* < 0.001). The ratio of satisfaction with life quality increased to 72.7% (16/22), 72.7% (16/22), and 68.2% (15/22) at 3, 6, and 12 mo, respectively. In total, 86.4% (19/22) subjects reduced their PPIs consumption after treatment. Of these, 22.7% (5/22), 31.8% (7/22), and 40.9% (9/22) subjects stopped medication use at 3 mo, 6 mo, and 12 mo, respectively (Table 3).

#### 3.2.2. Indicators of pH-Impedance Monitoring and Esophageal Manometry

Two common methods were used, the complete case method and carryforward method for managing missing values. In both of these methods, there were no differences in AET, LES pressure, and total reflux episodes between baseline and 12 mo after radiofrequency treatment, which indicated that the (antireflux effect of radiofrequency treatment might not contribute to the improvement of these symptoms (Table 4).

#### 3.2.3. Positive Cell Density of TRPV1 and CGRP

In order to comprehensively explore the relationship between the change in afferent fiber density in distal esophageal mucosa and radiofrequency treatment, we used 2 common methods, the complete case method and carryforward method for managing missing values. In both of these methods, radiofrequency ablation significantly decreased the positive cell density of both TRPV1 and CGRP at 12 mo after treatment (*P* < 0.001), which indicated that radiofrequency energy might effectively damage the afferent fibers and block visceral sensation in the distal esophagus (Table 5).

### 3.3. Correlation between Symptoms and Density of Afferent Fibers in the Distal Esophagus

**Figure 5:**
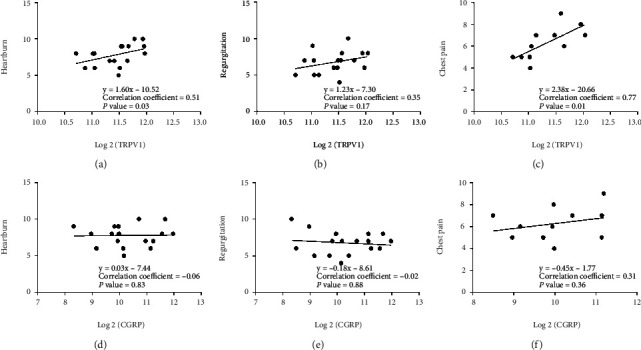
Correlations between symptoms and the positive cell density of TRPV1 and CGRP. TRPV1 was moderately and highly correlated with heartburn (a) and chest pain (c), but not regurgitation (b), respectively. However, CGRP was not significantly related to any symptoms (d–f).

In order to determine that damaging afferent fibers in the distal esophagus by radiofrequency ablation contribute to the improvement in symptoms, we further examined the relationship between symptoms and density of afferent fibers in the distal esophagus in reflux hypersensitivity patients. Before radiofrequency treatment, we found that the positive cell density of TRPV1, but not CGRP, was moderately and highly correlated with heartburn (*r* = 0.51, *P* = 0.03) and chest pain (*r* = 0.77, *P* < 0.01), respectively. These findings indicated that higher density of TRPV1+ afferent fibers could lead to more severe symptoms of heartburn and chest pain, which partially explained the refractory symptoms with no change in AET, LES pressure, and total reflux episodes in reflux hypersensitivity patients. In addition, the correlation tests also provided evidence that the improvement in symptoms after radiofrequency ablation was strongly associated with the downregulation of TRPV1 (Figure 5).

### 3.4. Complications

No major complications or deaths occurred during the study. Some patients experienced temporary minor complications, including throat discomfort (17 cases), transient nausea/vomiting (9 cases), retrosternal discomfort (7 cases), mild fever (5 cases), belching (3 cases), and transient dysphagia (1 case).

## 4. Discussion

Although seldom studied in reflux hypersensitivity, endoscopic radiofrequency ablation is one of the popular options for treating GERD. Several mechanisms have been proposed to explain radiofrequency ablation effects in GERD, including inhibition of transient LES relaxations and increasing LES pressure, and this might contribute to a decrease in reflux events [5–7]. However, a number of previous studies found that the obvious symptom relief in patients with typical GERD symptoms could not be explained by ambulatory or pH data [8, 9, 15–18]. A theory that radiofrequency can induce ablation of nociceptors and afferent fibers resulting in decreased esophageal sensitivity has been proposed. As peripheral sensitization appears to be one of the main underlying mechanisms of symptom generation in reflux hypersensitivity [19], this theory seemed worthy of further investigation in our study.

Although the reflux is “physiological”, it occurs predominately in the distal esophagus, so the procedure is performed near the gastroesophageal junction with reference to GERD. Among the 22 reflux hypersensitivity patients in the present study, 81.8%, 77.3%, and 50% complained of heartburn, regurgitation, and noncardiac chest pain, respectively, and at least two of these symptoms were present in each patient at baseline. The Likert score of these three typical symptoms decreased 3 to 12 mo after the procedure. In addition, improvement in satisfaction was evident at 3 mo after the procedure and was sustained over the entire observational period of 1 year. In agreement with most previous studies on reflux diseases [7, 16], we also found that radiofrequency ablation significantly changed PPI usage in reflux hypersensitivity patients, as 86% patients used half-dose PPI or even discontinued treatment 12 mo after radiofrequency ablation, with only 3 patients still requiring the regular or higher dose to control refractory symptoms.

This is the first study to evaluate the role of TRPV1 and CGRP in the distal esophageal mucosa of reflux hypersensitivity patients. TRPV1 and CGRP are often co-exist in the esophagus [20]; they are well-known biochemical markers of sensory afferent fibers [12, 14] and may play an important role in esophageal hypersensitivity [21–26]. The density of TRPV1 and CGRP was much lower 12 mo after radiofrequency ablation in our patients. Of note, TRPV1-positive cell density was strongly associated with chest pain and moderately associated with heartburn, which indicates the reduction of TRPV1 after radiofrequency ablation may at least play an essential role in relieving heartburn and chest pain in reflux hypersensitivity patients. Unexpectedly, the correlation coefficient showed an extremely poor association between CGRP-positive cell density and reflux hypersensitivity symptoms; we suspected that the downregulation of CGRP-positive cell density might only lead to a minor change accompanied by decreased TRPV1 after radiofrequency ablation.

The present study has some notable limitations that reduce the strength of our conclusions. First, the sample size in this single-center study was small, and the follow-up period was short. In addition, we did not include a sham-controlled group to eliminate the placebo effect. Furthermore, several patients were unable to undergo repeated invasive examinations such as ambulatory, 24-h pH-impedance monitoring, and esophageal biopsy during the follow-up period, which resulted in incomplete critical information when determining the mechanism of treatment. Further sham-controlled research on the long-term therapeutic values of radiofrequency ablation in a large population of reflux hypersensitivity patients is required.

In summary, our research first confirmed the value of endoscopic radiofrequency ablation in the management of reflux hypersensitivity regarding symptom relief, satisfaction, and PPIs consumption. The dramatically reduced TRPV1 and CGRP in the distal esophageal mucosal after radiofrequency ablation as well as the obvious correlation between TRPV1 and the severity of heartburn or chest pain suggested that altered TRPV1 in the distal esophagus may be an important pathophysiological factor and a therapeutic target in the treatment of reflux hypersensitivity.

## Figures and Tables

**Table 1:** Baseline characteristics.

Variable	Subjects
Male gender (*n*%)	14 (63.6%)
Age (mean ± SD)	50.0 (12.3)
BMI (mean ± SD)	21.9 (2.1)
Duration of disease (mean ± SD)	3.1 (1.8)
Heartburn (*n*%)	18 (81.8%)
Acid regurgitation (*n*%)	17 (77.3%)
Chest pain (*n*%)	11 (50%)
Average SI	65%
Average SAP	98%
Total reflux episodes (mean ± SD)	31.4 ± 3.1

**Table 2:** Severity of symptoms before and after treatment.

	3 mo		6 mo		12 mo	
	Change from baseline	*P*	Change from baseline	*P*	Change from baseline	*P*
Heartburn	-5.3 (1.1)	<0.001	-5.6 (1.3)	<0.001	-5.3 (1.3)	<0.001
Regurgitation	-4.4 (1.2)	<0.001	-4.8 (1.5)	<0.001	-4.9 (1.4)	<0.001
Chest pain	-3.5 (1.1)	<0.001	-3.7 (1.4)	<0.001	-3.5 (1.0)	<0.001

**Table 3:** Degree of satisfaction with life quality and PPI consumption before and after treatment.

	3 mo			6 mo		12 mo	
	Pretreatment	Posttreatment		Posttreatment	*P*	Posttreatment	*P*
Degree of satisfaction			<0.001		<0.001		<0.001
Dissatisfied	18 (81.8)	1 (4.5)		3 (13.6)		2 (9.1)	
Acceptable	4 (18.2)	5 (22.7)		3 (13.6)		5 (22.7)	
Satisfied	0 (0.0)	16 (72.7)		16 (72.7)		15 (68.2)	
PPI consumption			<0.001		<0.001		<0.001
Regular dose	22 (100)	3 (13.6)		3 (13.6)		3 (13.6)	
Half dose	0 (0.0)	14 (63.6)		12 (54.5)		10 (45.5)	
No medication	0 (0.0)	5 (22.7)		7 (31.8)		9 (40.9)	

PPI: proton pump inhibitor.

**Table 4:** Absolute differences in AET and LES pressure at baseline and 12 mo after treatment.

	Subjects with complete data	Absolute change from baseline	*P*	Carrying last value forward	Absolute change from baseline	*P*
AET	15	-0.1 (-1.2,1.1)	0.755	22	0.0 (-0.5,0.4)	0.755
LES pressure	15	1.9 (-0.6,4.4)	0.121	22	0.0 (0.0,3.2)	0.118
Total reflux episodes	15	-3.9 (-1.1,1.3)	0.677	22	-3.5 (-1.2,1.3)	0.679

AET: acid exposure time; LES, lower esophageal sphincter.

**Table 5:** Absolute differences in the number of afferent fibers in distal esophageal mucosa at baseline and 12 m after treatment.

	Subjects with complete data	Absolute change from baseline	*P*	Carrying last value forward	Absolute change from baseline	*P*
TRPV1	18	-914.5 (-1103.8, -760.2)	<0.001	22	-844.5 (-1067.2, -309.8)	<0.001
CGRP	18	-546.0 (-688.2, -227.0)	<0.001	22	-474.0 (-675.0, -28.8)	<0.001

Complete case analysis evaluated only subjects with complete data at both baseline and 12 mo. Carrying last value forward analysis evaluated all patients after carrying forward the baseline value for subjects with missing 12-mo data.

## Data Availability

We can provide our data on request; if it is necessary, you can contact and email jyxqrh@163.com then.
